# A rare case of metastatic renal carcinoid

**DOI:** 10.1186/1471-2490-10-22

**Published:** 2010-12-14

**Authors:** Yoshiharu Kato, Kogenta Nakamura, Yoshiaki Yamada, Genya Nishikawa, Takahiko Yoshizawa, Kenji Zennnami, Remi Katsuda, Motoi Tobiume, Shigeyuki Aoki, Tomohiro Taki, Nobuaki Honda

**Affiliations:** 1 Department of Urology, Aichi Medical University School of Medicine, Nagakute, Aichi 480-1195, Japan

## Abstract

**Background:**

Carcinoid is an endocrine cell tumor with low-grade atypia, which is generally a low-grade malignant cancer with a good prognosis. Metastatic renal carcinoid is even rarer than primary carcinoids.

**Case presentation:**

We present our experience of a patient with metastatic renal carcinoid from the gastrointestinal tract.

**Conclusions:**

The carcinoid tumor of the kidney in our patient, who had a history of liver metastasis from rectal carcinoid, was considered metastatic based on the pathological findings.

## Background

Carcinoid tumor was first detected as multiple tumors in the ileum by Lubarsch during autopsy more than 100 years ago [1]. The concept of this disease is an endocrine cell tumor with low-grade atypia, which is generally a low-grade malignant cancer with a good prognosis. About 70% of carcinoid tumors occur in the gastrointestinal tract, including the rectum, appendix, stomach, and ileum. The rest of them arise mainly from neuroendocrine cells in the pancreas, gonads and pulmonary bronchi, and they rarely occur in the kidney. Metastatic renal carcinoid is even rarer, with only one reported case [2]. Here, we present our experience of a patient with metastatic renal carcinoid with a review of the literature.

## Case presentation

A 56-year-old man underwent resection of a rectal carcinoid twelve years ago, and subsequently underwent resection of liver metastases of rectal carcinoid twice, twelve and ten years ago. Two years ago, he was found to have a 3-cm mass in the left kidney on ultrasonography during a general checkup, and attended our department. Results of routine blood chemical analysis and urinalysis were unremarkable.

**Figure 1:**
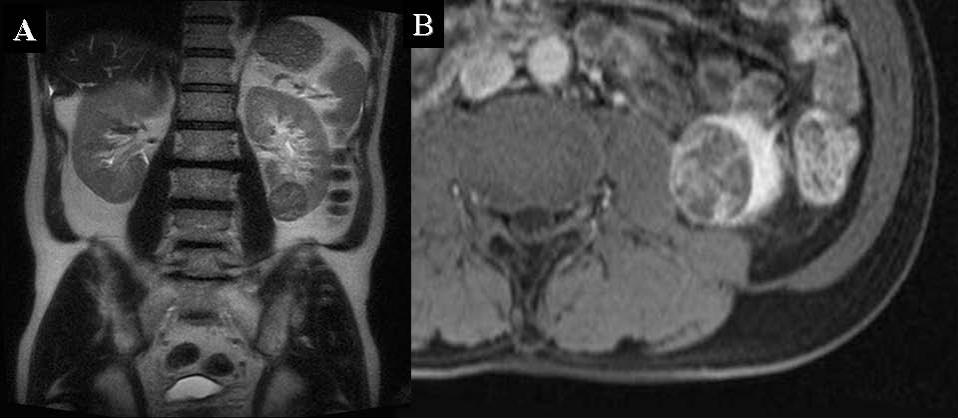
**CT images**. (A): A 3 cm poorly enhanced, well-defined mass was observed in the inferior pole of the left kidney on CT. (B): A 2 × 2 × 3 cm well-defined, solid tumor with a white capsule was detected in the inferior pole of the left kidney at operation. Inside the mass, hematoma and necrotic tissue were partly observed. Neither obvious enlargement of lymph nodes nor metastasis to intraperitoneal organs was seen.

Ultrasonography revealed a 3-cm relatively well-defined mass in the lower pole of the left kidney, and the interior of the mass was low echoic, unclear of margin, and hypovascular. Contrast-enhanced abdominal CT demonstrated a poorly enhanced, well-defined mass at the above-mentioned site in both the arterial and venous phases (Figure 1A). MRI showed a well-defined mass containing irregular high-signal-intensity areas on T1-weighted images and relatively low-signal-intensity areas on T2-weighted images. Bone scintigraphy did not reveal any obvious bone metastases.

Since these findings did not rule out malignancy, left renal cell carcinoma T1aN0M0 was diagnosed. According to the patient's strong wishes, left nephrectomy was performed laparoscopically through the retroperitoneal approach. Examination of the resected specimen revealed a 2 × 2 × 3 cm well-defined, solid tumor with a white capsule in the inferior pole of the kidney (Figure 1B).

**Figure 2:**
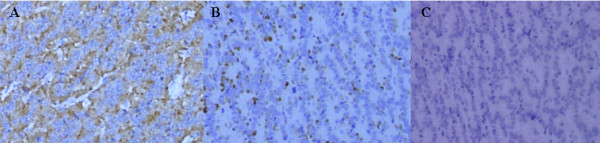
**Histopathological examination**. Immunostaining was positive for synaptophysin (A) and chromogranin A (B), and negative for p53 (C).

Histopathologically, hematoxylin and eosin staining revealed cells uniformly arranged in a trabecular or funicular pattern. The cytoplasm was ill-defined and showed eosinophilic staining. The nucleus was ovoid to short fusiform, with no obvious mitoses. The tissue was partly necrotic. Immunostaining was positive for synaptophysin and chromogranin A, and negative for p53 (Figure 2A, B, C). These findings were consistent with those of hematoxylin and eosin staining of specimens of the rectum and liver reported by the patient's former physician. Electron microscopy was not performed. Based on the pathological findings and the history of liver metastasis from rectal carcinoid, we diagnosed metastatic renal carcinoid.

Postoperatively, blood tests, urinalysis, imaging, gastrointestinal endoscopy and other examinations showed no findings suggestive of recurrence of carcinoid while two years.

## Discussion

Carcinoid tumors are generally considered to arise from neuroendocrine cells, and secrete different substances depending on their primary site. Such tumors characteristically secrete serotonin, although some of them are inactive. Accordingly, urinary secretion of 5-hydroxyindole acetic acid (5-HIAA), a metabolite of serotonin, increases. Other hormones and biochemical substances secreted from carcinoid tumors include corticotropin, histamine, dopamine, substance P, neurotensin, prostaglandins and kallikrein. Secretion of these vasoactive substances, including serotonin, may cause symptoms of palpitations, hot flushes, wheezing, diarrhea, and skin flushing, as well as carcinoid syndrome such as right-sided valvular disease. Since serotonin and other similar substances are metabolized mainly in the liver, carcinoid syndrome is observed only when such substances flow directly into the systemic circulation from metastatic lesions in the liver, lung, gonads etc., without involving the liver. From the perspective of a histogenetic pathway, carcinoid is a benign tumor originating from epithelial stem cells, and highly malignant carcinoids are referred to as endocrine cell carcinoma. For differentiation of malignancy, immunostaining for p53 is useful. Nishikura *et al*. conducted a study in a total of 98 patients with gastric endocrine cell carcinoma or carcinoid tumor, and reported that patients with carcinoid tumor did not have any p53 protein overexpression or genetic mutation [3].

Moyana *et al*. investigated the association between differentiation of gastrointestinal carcinoid and MIB-1, p53, and bcl-2 in 58 patients, and reported that MIB-1 and p53 could be prognostic factors [4].

Nearly 100 cases of primary renal carcinoid have been reported since Resnick *et al*. reported the first case in 1966 [5]. There has been no established view on factors involved in the occurrence of renal carcinoid, but the following mechanisms have been hypothesized: 1) Neuroendocrine cells that do not normally exist in the kidney undergo neoplastic changes, 2) stem cells induce neuroendocrine differentiation, 3) the transitional epithelium of the renal pelvis develops intestinal metaplasia, and renal carcinoid originates from neuroendocrine cells present at this site, and 4) renal carcinoid arises from the migration of intestinal or bronchial epithelium into the kidney [6].

Romero *et al*. reviewed 56 case reports of renal carcinoid [7]. The median age of the patients was 49 years, with no sex difference. Horseshoe kidney was present in 17.8% of patients, and renal carcinoid was incidentally diagnosed in 28.6% of patients. The most common symptom was abdominal pain, and carcinoid syndrome was seen in 12.7% of patients. Many (73.6%) of the tumors were 4 cm or more in diameter, and as many as 45.6% of the patients already had metastasis at initial diagnosis. Although lymph node metastases were detected in as many as 47% of patients, renal carcinoid was found to have a relatively good outcome, with a mean disease-free interval of 43 months during follow-up. However, some of the patients with metastasis to solid organs such as the liver, contralateral kidney or bone had a poor outcome and died within a few months. Krishnan *et al*. reported that renal carcinoid often complicates horseshoe kidney, polycystic kidney or renal teratomata [8]. In particular, horseshoe kidney is known to be associated with a 62% higher risk of carcinoid; such carcinoids tend to arise from a solid teratoma, and are considered to have a better outcome than those complicating other underlying renal diseases. Renal carcinoid is mostly detected incidentally by imaging, and is rarely accompanied by symptoms such as flank pain, hematuria or a palpable mass. Imaging findings of renal carcinoid are nonspecific, while angiography often reveals a hypovascular mass, and ultrasonography, CT, and MRI visualize a well-defined mass. This disease should be differentiated most importantly from renal cell carcinoma, as well as from benign renal tumors including oncocytoma, angiomyolipoma or malacoplakia. As in cases of other carcinoid, surgical resection is the mainstay of treatment for renal carcinoid. Kobayashi *et al*. reported the first case of renal carcinoid treated by laparoscopic partial nephrectomy [9].

To our knowledge, there has been only one reported case of metastatic renal carcinoid (metastasis from the lung to the right kidney) by Tal *et a l *[2]. This patient was a 64-year-old woman who underwent resection of a pulmonary carcinoid and then was incidentally found to have a renal carcinoid 2 years later during detailed investigation of a left neck mass. Abdominal CT revealed a hepatic mass and a renal mass measuring 5 × 8 cm. The renal mass had invaded the renal pelvis and vein. The patient did not have any other noteworthy abnormalities or signs of carcinoid syndrome. She underwent partial hepatectomy and right nephrectomy. Microscopically, immunostaining was positive for chromogranin and neuron-specific enolase. Indium-111 octreotide scan was performed and showed positivity along with the left neck mass. The somatostatin analog octreotide, which is expressed in 80% or more of carcinoid tumors, was found to be useful for diagnostic staging. But it's impossible to use octeride at our institution.

In this case, we should choose and recommend laparoscopic partial nephrectomy even if the patient had strong wish to laparosopic radical nephrectomy. Because, Simmons *et al*. reported that in appropriate patients with Stage T1b-T3 tumor >4 cm, laproscopic partial nephrectomy provides equivalent oncologic efficacy and superior renal functional outcomes compared with laparoscopic radical nephrectomy [10].

## Conclusions

The carcinoid tumor of the kidney in our patient, who had a history of liver metastasis from rectal carcinoid, was considered metastatic based on the pathological findings. In view of the tumor being p53-negative and no apparent recurrence to date, he seemed to have a good prognosis.

## Competing interests

The authors declare that they have no competing interests.

## Authors' contributions

YK drafted the report, and approved the final version of the manuscript.

YY, GN, TY, KZ, RK, MT, SA, TT and NH cared for the patient and approved the final version of the manuscript.

KN drafted the report, cared for the patient and approved the final version of the manuscript.

## Authors information

Department of Urology, Aichi Medical University School of Medicine, Nagakute, Aichi 480-1195, Japan.

## Pre-publication history

The pre-publication history for this paper can be accessed here:

http://www.biomedcentral.com/1471-2490/10/22/prepub
